# The Effect of Dietary Leucine Supplementation on Antioxidant Capacity and Meat Quality of Finishing Pigs under Heat Stress

**DOI:** 10.3390/antiox11071373

**Published:** 2022-07-15

**Authors:** Yunju Yin, Yating Liu, Geyan Duan, Mengmeng Han, Saiming Gong, Zhikang Yang, Yehui Duan, Qiuping Guo, Qinghua Chen, Fengna Li

**Affiliations:** 1 College of Animal Science and Technology, Hunan Agricultural University, Changsha 410128, China; yinyj1124@163.com (Y.Y.); liuyt0129@163.com (Y.L.); 15197442484@163.com (S.G.); yangzhikang2021@163.com (Z.Y.); 2 Hunan Provincial Key Laboratory of Animal Nutritional Physiology and Metabolic Process, Key Laboratory of Agro-Ecological Processes in Subtropical Region, Institute of Subtropical Agriculture, Chinese Academy of Sciences, Hunan Provincial Engineering Research Center for Healthy Livestock and Poultry Production, Changsha 410125, China; duangeyan21@mails.ucas.ac.cn (G.D.); 15591808189@163.com (M.H.); duanyehui@isa.ac.cn (Y.D.); lifengna@isa.ac.cn (F.L.); 3 University of Chinese Academy of Sciences, Beijing 100049, China

**Keywords:** leucine, antioxidant, growing-finishing pigs, meat quality

## Abstract

This study examined the effects of dietary leucine supplements on antioxidant capacity and meat quality in growing-finishing pigs. A total of 24 crossbred (Duroc × Landrace × Yorkshire) pigs with an average initial weight of 68.33 ± 0.97 kg were randomly allotted to three treatment groups. All pigs were exposed to constant heat stress. Each group of pigs was fed a basal diet, or a diet supplemented with increasing levels of leucine (0.25% or 0.50%). The results showed that leucine intake could improve average daily gain and reduce feed/gain of finishing pigs under heat stress (*p* < 0.05). The supplementation of leucine could improve the carcass slant length (*p* = 0.09), and dramatically increased loin-eye area of the finishing pigs (*p* < 0.05) but had no significant effect on other carcass traits. Compared with the control group, 0.50% leucine markedly reduced drip loss and shear force of *longissimus dorsi* muscle, and increased pH value at 24 h after slaughter (*p* < 0.05). Dietary supplementation of 0.25% leucine increased the contents of inosine monophosphate and intramuscular fat in *biceps femoris* muscle (*p* < 0.05). Supplementation of 0.25% or 0.50% leucine significantly stimulated the activities of antioxidant enzymes while reduced the level of MDA in serum, liver and *longissimus dorsi* muscle (*p* < 0.05). Compared with the control group, 0.50% leucine supplementation markedly modulated the relative mRNA expression levels of genes related to muscle fiber type and mitochondrial function in *longissimus dorsi* muscle and the gene relative antioxidant in the liver (*p* < 0.05). In conclusion, dietary leucine supplementation could improve the growth performance and meat quality of the finishing pigs under heat stress, and the pathway of Keap1-NRF2 and PGC-1α-TFAM might be involved.

## 1. Introduction

Predictive environmental models indicate that environment-induced hyperthermia will continue to threaten human and animal health, and that the percentage of morbidity and mortality will increase as global temperatures are expected to rise [1,2,3]. Data from the Food and Agriculture Organization of the United Nations (FAO) show that more than 50% of better pig production is located in tropical and subtropical climates. According to Burton et al. [4], the optimum temperature for growing-finishing pigs varies between 16 °C and 20 °C, respectively. High ambient temperatures and humidity are a challenge to animal production around the world and one of the major causes of economic and production losses in pig production [5]. The economic and production losses caused by heat stress are mainly due to reduced meat production and fertility [6,7]. Typically, high temperatures reduce feed intake in finishing pigs, which limits energy intake and thus decreases intramuscular fat deposition [8,9,10]. High ambient temperatures lead to more exudative pig meat, lower water holding capacity, and greater shear force, along with reduced pH value and lighter color, which adversely affects the nutritional properties and tenderness of the meat [8]. 

Leucine, chemically known as α-amino isophanic acid, is an essential amino acid and branched chain amino acid (BCAA), widely found in animal protein and milk, eggs, pork, beef, chicken and other dairy products, as well as beans [11,12,13]. Leucine has a strong oxidative capacity and its main physiological functions include regulating protein metabolism [14] and providing oxidation energy [15]. This energy supply can be used for specific physiological periods such as hunger, breastfeeding, stress and exercise [16]. The team’s previous work focused on the regulatory role of leucine in lipid metabolism [17,18]. The effects of leucine on the pork quality and antioxidant properties of growing-finishing pigs under high temperature have not been investigated, and the current research on the improvement of antioxidant capacities of leucine is only limited to piglets. Therefore, this study was conducted to investigate the effects of different leucine concentrations on growth performance, meat quality and antioxidant capacities of finishing pigs at high temperature to provide a basis for the addition and usage of leucine in finishing pigs.

## 2. Materials and Methods

### 2.1. Animal and Experimental Design

The animal experiments were approved by the Committee on Animal Care of the Institute of Subtropical Agriculture, Chinese Academy of Sciences. Animal testing meets the requirements of animal ethics and animal welfare.

Twenty-four healthy growing pigs (Duroc × Landrace × Yorkshire) with a body weight of 68.33 ± 0.97 kg were randomly divided into three groups with eight replicates (pens) per group and one pig per replicate according to their body weight. These pigs in the experiment are all boars. The adaptive feeding experiment was conducted for 3 days and then the formal experiment was started. Each pig was fed in a single pen. The length of this pen is 2 m, the width is 1.5 m, the floor is leaky seam floor, all the pigs are free to move around inside. The experimental diet was a corn-soybean meal basal diet, which was designed to meet NRC (2012) standards, with protein level about 14%. The ingredients and nutritional composition of basal diet was shown in Table 1. Experimental diets were as follows: (1) basal diet (control group); (2) basal diet + 0.25% leucine (0.25% Leu group); (3) basal diet + 0.50% leucine (0.5% Leu group). All pigs were given free access to feed and water during the 42-day trial period. Feed intake for each pig was recorded daily to determine average daily feed intake (ADFI), average daily gain (ADG), and feed/gain (F/G). All pigs were weighed at the beginning and end of the experiment.

Electronic temperature and humidity recorder (model: Kth-350-i, Bordeaux, France) was hung in the experimental barn about 1.5 m above the ground, and there were 3 houses in average. Temperature and humidity were automatically recorded every 0.5 h during the whole experiment period. Temperature-humidity index (THI) is usually used to describe whether and the degree of heat stress in the process of livestock and poultry breeding.

THI = 0.8 Ta + RH/100 × (Ta − 14.4) + 46.4

Ta (°C) is the ambient temperature; RH (%) indicates the relative humidity.

**Figure 1:**
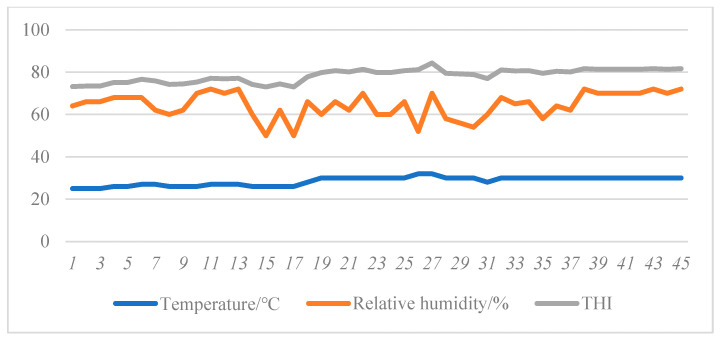
Temperature and humidity in the piggery during the experimental period.

Continuous changes of ambient temperature and humidity in piggery during the experiment period are shown in Figure 1. The highest temperature in the piggery is 30 °C, the lowest temperature is 25 °C; The relative humidity in the piggery varies from 50% to 70%. The THI curve showed that the range of THI was 74.16~84.32 in the formal trial period. THI can be classified into certain heat stress thresholds: THI < 74 indicates a comfortable environment, 74 ≤ THI < 78 indicates mild heat stress, 78 ≤ THI < 82 indicates moderate heat stress, and THI ≥ 82 indicates severe heat stress [19]. During the whole experiment period, and the finishing pigs were in heat stress state.

### 2.2. Sample Collection

At the end of the trial, all the pigs were fasted overnight and harvested by electrical stunning followed by exsanguination, blood samples were collected into plain tube and placed at room temperature for 30 min, then centrifuged at 3000× *g* for 10 min at 4 °C. Serum was collected and stored at −80 °C for further analysis [20]. Liver sample was frozen in liquid N_2_ and stored at −80 °C for subtest. After weighing the carcass, muscle samples of *longissimus dorsi* muscle and *biceps femoris* muscle were quickly removed from the right side of the carcass. A portion of the *longissimus dorsi* muscle and *biceps femoris* muscle samples were immediately frozen in liquid N_2_ and then stored at −80 °C until further analysis.

### 2.3. Carcass Traits Analysis

Weigh the left side of the carcass and measure the length and better backfat thickness of the carcass. Carcass length was recorded as the distance from the superior margin of the symphysis pubis to the junction of the first rib and sternum. The backfat thickness between the sixth and seventh ribs was measured with vernier calipers, the width and height of the *longissimus dorsi* cross section were measured with vernier calipers, and the loin-eye area was calculated (width × height × 0.7). The left side of the carcass is then dissected into skeletal muscle, fat, bone and skin. The carcass composition is calculated by dividing the anatomical tissue by the carcass weight on the left. Slaughter rate is calculated by dividing carcass weight by slaughter weight × 100%. Next, a split was performed at the sixth-seventh rib on the left side of the cadaver to determine the *longissimus dorsi* muscle area. Weighed and calculated the total fat rate percentage and lean mass percentage.

### 2.4. Determination of Meat Quality

The *longissimus dorsi* muscle and *biceps femoris* muscle samples were cut after slaughter. The mean pH was obtained at 45 min and 24 h with the aid of knife-type electrode pH meter (PH-STAR, MATTHAUS, Germany), with measurements made at three points along the same sample after defrosting. For color measurement, 2.5 cm thick sections were exposed to air for 45 min and 24 h for reaction of myoglobin with atmospheric oxygen. The staining measurements were obtained using a colorimeter (Chroma Meter CR410, Konica Minolta Inc.^®^, Osaka, Japan) apparatus operating in the CIE system, evaluating values of L* (lightness), a* (redness) and b* (yellowness) of the meat [21].

The cooked samples of *longissimus dorsi* muscle were cut into cubes (1.0 × 1.0 × 2.0 cm^3^) and subjected to the texture analyzer apparatus (TMS-PRO, FTC, United Kingdom), with the blade moving at 1.5 mm/s downward using 40 g force. The equipment program generated stress vs. time curves, determining the shear force, expressed in N [22], eight replicates of each sample were measured to calculate the average value. The drip loss was determined at 24 h after slaughter. Briefly, the long length of a muscle section was cut along the fiber direction (2 cm × 3 cm × 5 cm), trimmed of fat, weighed, suspended in a polyethylene plastic bag, ensuring that the sample did not make contact with the bag and then stored at 4 °C, After 24 h, the sample was reweighed to calculate drip loss percentage.

### 2.5. Muscle Chemical Composition

Samples of *longissimus dorsi* muscle and *biceps femoris* muscle were freeze-dried for 72 h, and then the ceramic plates were reweighed. The difference between the initial and dried ceramic plate weights was used to calculate the percentage of moisture. Dried muscle samples were subsequently pulverized using a hammer mill, and analyzed for protein and intramuscular fat content (IMF) according to AOAC (2005) methods [23].

Contents of inosinic acid were determined using a High-Performance Liquid Chromatography method. Briefly, 0.2 g freeze-dried muscle sample powder was added to 5 mL perchloric acid (HClO4, 0.6 mol/L), and the mixture was homogenized with an ice bath in an ultrasonic cleaning machine. Afterward, the mixture was centrifuged (6000× *g*, 10 min, 4 °C) for deproteinization. The volume of supernatant was then made up to 10 mL with double-distilled water. Half of the mixture was adjusted to pH 6.9–7.1 using a potassium hydroxide (KOH, 1 mol/L) and perchloric acid (HClO4, 0.6 mol/L) solution, allowed to stand for 10 min in an ice bath, and filtered through a 0.22 mm microfilter to remove potassium perchlorate. The filtrate was adjusted to a total volume of 10 mL with distilled water, and it was analyzed by high-performance liquid chromatography (1260 Infinity II, Agilent, Santa Clara, CA, USA). The chromatographic conditions were as follows: the reversed-phase chromatographic column (Agilent ZORBAX Eclipse XDB-C18; 4.6 mm × 250 mm, 5 μm), with a mobile phase of phosphate buffer solution (100%) at a flow rate of 1.0 mL/min, was used in this chromatographic assay. The injection volume, column temperature, and UV detection wavelength were 10 μL, 25 °C, and 210 nm, respectively.

### 2.6. Serum, Liver and Longissimus Dorsi Muscle Antioxidant Enzyme Activity

The liver and *longissimus dorsi* samples were ground thoroughly in a mortar with liquid N_2_, and put into an EP tube with grinding beads, DEPC water and protease inhibitor. The thoroughly mixed with a homogenizer. After that, the supernatant was centrifuged to make tissue homogenate. The activities of total superoxide dismutase (T-SOD), glutathione peroxidase (GSH-Px), catalase (CAT), total antioxidative capacity (T-AOC), and the contents of malondialdehyde (MDA) were assayed using colorimetric methods with a Microplate Reader (Infinite M200 PRO, TECAN, Männedorf, Switzerland). The assays were conducted with the commercial kits purchased from Changsha Aoji Biotechnology Co., Ltd., (Changsha, China) and their corresponding procedures.

### 2.7. Total RNA Isolation and Quantitative Real-Time PCR Analysis

Total RNA isolation and real-time quantitative PCR were conducted as previously described [18]. In brief, total RNA was extracted from liver and *longissimus dorsi* samples using Trizol reagent (Hunan Aikerui Bioengineering Co., Ltd., Changsha, China). The purity of the total RNA was verified using a NanoDrop ND2000 (NanoDrop Technologies Inc., Wilmington, DE, USA) at 260 and 280 nm, the OD260/OD280 ratios of the RNA samples were all between 1.8 and 2.0. The total RNA was treated with DNase I (Hunan Aikerui Bioengineering Co., Ltd., Changsha, China) to remove DNA and reverse transcribed to complementary deoxyribonucleic acid (cDNA) using Evo M-MLV RT Kits with gDNA clean for qPCR (Hunan Aikerui Bioengineering Co., Ltd., Changsha, China) following the manufacturer’s protocol. Quantitative real-time PCR was performed using an ABI 7900 HT real-time PCR system (Applied Biosystems, Branchburg, NJ, USA) with SYBR Green Premix Pro Taq HS qPCR Kits (Hunan Aikerui Bioengineering Co., Ltd., Changsha, China). The PCR system consisted of 5 μL SYBR Green Pro Taq HS Premix, 2 μL cDNA, 2.2 μL RNase free water and 0.4 μL primer pairs (forward and reverse) in a total volume of 10 μL. The PCR protocols included one cycle at 95 °C for 30 s, 40 cycles at 95 °C for 5 s and 60 °C for 30 s. Glyceraldehyde-3-phosphate dehydrogenase (GAPDH) was used as the endogenous control gene to normalize the expression of target genes according to the comparative Ct method as follows: 2^−ΔΔCt^ (ΔΔCt = ΔCt _gene of interest_ − ΔCt_GAPDH_) [24]. Primer sequences are shown in the Table 2.

### 2.8. Statistical Analysis

All experimental orthogonal data were tested by variance homogeneity, and then analyzed using one-way analysis of variance (ANOVA) of SPSS (version 26.0, SPSS Inc., Chicago, IL, USA), and then Duncan multiple comparison test was performed. Results were expressed as mean and SEM, *p* < 0.05 was considered significant, and 0.05 ≤ *p* < 0.10 was considered as trend.

## 3. Results

### 3.1. Growth Performance

Table 3 shows the effects of different leucine supplemental levels on the growth performance of growing-finishing pigs. There were no significant differences in initial body weight, final body weight and ADFI among the three groups (*p* > 0.05), compared with control group, ADG and F/G in treatment group were observably increased (*p* < 0.05), and the ADFI reached the maximum in the 0.25% group, but there was no significant difference between the 0.50% group and the 0.25% group, which had the lowest F/G.

### 3.2. Carcass Traits

Table 4 shows that no conspicuous differences were observed between the three groups in carcass weight, slaughter rate, carcass percentage, carcass straight length, lean mass percentage and fat percentage (*p* > 0.05). Compared with the control group, the loin-eye area in the 0.50% Leu treatment group was significantly increased (*p* < 0.05). In addition, both 0.25% and 0.50% Leu treatment groups tended to improved carcass traits of slant length (0.05 < *p*<0.1), and notably decreased backfat thickness (*p* < 0.05) compared with the control group.

### 3.3. Meat Quality

As shown in Table 5, compared with the control group, the drip loss and sheer force of *longissimus dorsi* muscle in groups 0.25% and 0.50% Leu treatment were notably reduced (*p* < 0.05). In terms of meat color, leucine supplementation significantly reduced pH value of *longissimus dorsi* muscle 24 h after slaughter (*p* < 0.05) but had no significant effect at 45 min (*p* > 0.05). No significant differences were observed in L*, a * and b * color values at either 24 h or 45 min compared with the control group (*p* > 0.05).

### 3.4. Chemical Composition of the Muscle Tissue

Table 6 shows that no significant effects of leucine on CP, IMF and IMP contents of *longissimus dorsi* muscle were observed (*p* > 0.05). However, compared with the control group, the contents of IMF and IMP in the treatment group of 0.25% Leu treatment group were significantly increased in *biceps femoris* muscle (*p* < 0.05).

### 3.5. Antioxidant Capacity

According to the data in Table 7, in serum, compared with the control group, 0.50% leucine could significantly increase the activities of T-AOC, SOD and CAT (*p* < 0.05), significantly decreased MDA content (*p* < 0.05). However, no significant effect of leucine supplementation on GSH-Px activity was found (*p* > 0.05). As shown in Table 8, in liver tissue, compared with the control group, the activities of GSH-Px and T-AOC were significantly increased in both 0.25% and 0.50% groups (*p* < 0.05). Table 9 showed that in *longissimus dorsi* muscle tissue, compared with the control group, the activity of T-AOC was increased, and the content of MDA was decreased in both 0.25% and 0.50% groups.

### 3.6. Relative mRNA Expression Levels of the Key Genes

**Figure 2:**
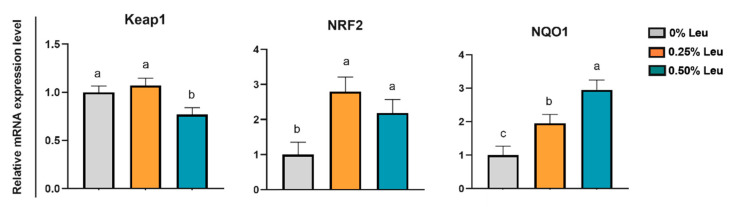
Relative mRNA expression levels of key genes related to antioxidant capacity in liver. Leu: Leucine. ^a–c^ Different superscript letters on the same line are significant differences (*p* < 0.05).

**Figure 3:**
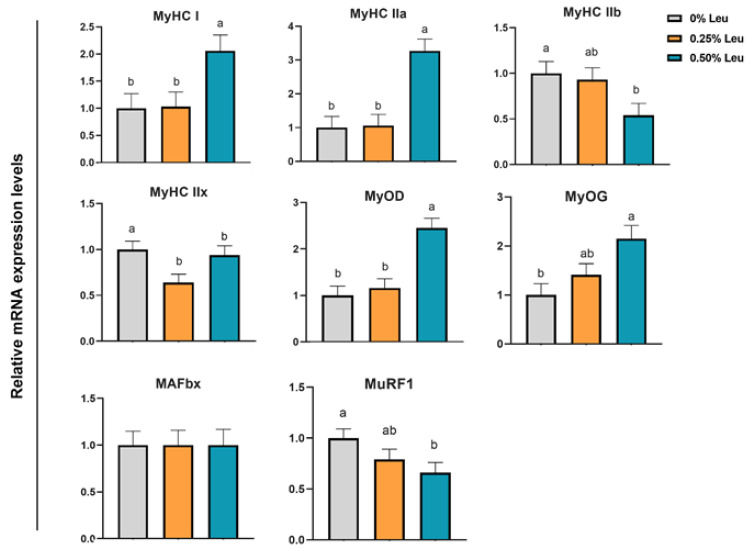
Relative mRNA expression levels of f key genes related to muscle fiber growth in *longissimus dorsi* muscle. Leu: Leucine. ^a,b^ Different superscript letters on the same line are significant differences (*p* < 0.05).

**Figure 4:**
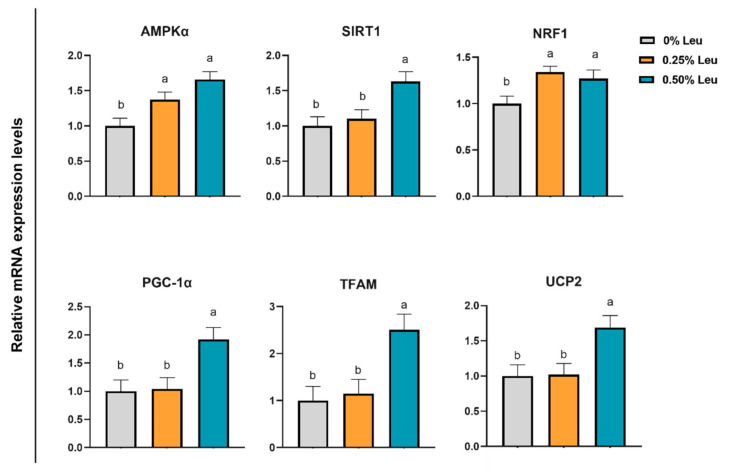
Relative mRNA expression levels of key genes related to mitochondrial function in *longissimus dorsi* muscle. Leu: Leucine. ^a,b^ Different superscript letters on the same line are significant differences (*p* < 0.05).

As shown in Figure 2, supplementation of 0.25% Leu and 0.50% Leu significantly increased the relative mRNA expression levels of NRF2 and NQO1 (*p* < 0.05), and 0.50% leucine markedly decreased the relative mRNA expression level of Keap1 in liver (*p* < 0.05). Figure 3 shows, in *longissimus dorsi* muscle, relative mRNA expression levels of MyHC IIb and MyHC IIx related to muscle fiber conversion in 0.25% and 0.50% groups were significantly downregulated (*p* < 0.05), while mRNA expression levels of MyHC IIa and MyHC I were significantly increased (*p* < 0.05). Additionally, 0.50% leucine could up-regulated the relative mRNA expression levels of MyoG and MyOD (*p* < 0.05), and decreased the relative mRNA expression levels of MuRF1 (*p* < 0.05) related to muscle fiber growth, but it had no effect on relative mRNA expression levels of MAFbx (*p* > 0.05). As shown in Figure 4, compared with the control group, the relative mRNA expression levels of TFAM, UCP2, NRF1, AMPKα, PGC-1α and SIRT1 related to mitochondrial function in *longissimus dorsi* muscle were higher in the treatment group (*p* < 0.05).

## 4. Discussion

Leucine is an essential amino acid that cannot be synthesized by animals. Leucine can promote protein synthesis by activating mammalian target of rapamycin (mTOR) signaling pathway, maintaining muscle mass, inhibiting fat synthesis, promoting fat decomposition, and increasing energy expenditure [25] Pigs are highly susceptible to ambient temperature due to the lack of functional sweat glands and a relatively thick layer of subcutaneous fat. The results showed that in high temperature and a high humidity environment, dietary leucine significantly increased the ADG and significantly reduced the F/G of finishing pigs but had no significant effect on other growth performance indexes. These results are consistent with the research of Madeira et al. [12]. Similarly, a previous study showed that dietary leucine had no significant effect on body weight of C57BL/6 mice [26]. Heat stress leads to changes in intestinal microbiota and affects gastrointestinal digestion and absorption [27]. Studies have found that with the increase of leucine dose, *Firmicutes–Bacteroidetes* ratio gradually decreased, the possible reason is that 0.50% leucine decreased the beneficial and dominant bacteria such as *Firmicutes* and *Bacteroidetes* in intestinal, increased leucine is closely associated with weight loss [28]. In this study, supplementation of leucine could significantly reduce the F/G of finishing pigs, which may be that leucine alleviates intestinal damage caused by heat stress and improves the rate of digestion and absorption of nutrients [29].

Slaughter rate, average backfat thickness, loin-eye area, lean mass percentage and total fat percentage are important indicators to reflect the carcass quality. In this study, the carcass slant length, backfat thickness and loin-eye area of the finishing pigs treated with 0.50% leucine were improved. Leucine may decrease backfat thickness by SIRT1–AMPK–PGC-1α axis [30]. Leucine supplementation could alleviate the phenomenon of high drip loss and shear force caused by heat stress. The increase in the abundance of MyHC-I, which contributes to increase water holding capacity and tenderness [31]. Hence, leucine might improve muscle water holding capacity and tenderness through improving muscle fibers composition. Under heat stress, anaerobic glycolysis accelerates the generation of energy (ATP) by decomposing muscle glycogen, accelerates the production of lactic acid, and leads to a rapid decrease in pH value [32]. Therefore, the addition of leucine in the diet could increase the pH value of *longissimus dorsi* muscle 24 h after slaughter. The possible reason is that leucine inhibits the glycolysis process in muscle and reduces the rate of pyruvate producing lactic acid [33], but did not affect other meat quality indexes, as reported by Madeira et al. [12], that dietary leucine had no significant change in cooked meat rate and meat color after slaughter.

The flavor of meat was positively correlated with substances such as IMF, IMP and inorganic salts [34]. Among them, IMP is an important basis of pork umami and can be used as an important index to evaluate pork flavor [35]. IMF content was positively correlated with tenderness, the more IMF, the better the tenderness after cooking [36]. This study showed that 0.50% leucine could significantly increase the contents of IMF and IMP in the *biceps femoris* muscle of growing-finishing pigs, which was also confirmed by Madeira et al. [12]. We hypothesize that the possible reason for this result is that leucine might promote the β-oxidation of fatty acid and inhibit glycolysis. Specifically, leucine supplementation under heat stress rapidly activates the activity of ATPase and promotes the decomposition of surplus ATP into ADP. Due to the activity of various enzymes, IMP is produced and further hydrolyzed to form hypoxanthine and ribose [37]. Thus, our data indicated that leucine supplementation could improve meat quality by increasing the contents of IMF and IMP under heat stress.

The long-term chronic heat stress caused by high temperature in summer will consume a large number of antioxidants in the body, damaging the body’s antioxidant defense system, leading to the decline of antioxidant enzyme activity. Reactive oxygen species (ROS) cannot be removed in time, resulting in the imbalance between oxidation and anti-oxidation, causing oxidative stress in the body [38]. The content of lipid peroxidation products and the activity of antioxidant enzymes can be used as the main indicators of oxidative stress. In the present experiment, 0.25% leucine can reduce MDA content in serum, liver and *longissimus dorsi muscle*. The findings are in line with the result of Huang et al. [39], which showed that 0.35% leucine treatment reduced jejunum MDA content in IUGR piglets.

Mammalian cells have a complex network of antioxidant enzymes (such as GPx and SOD) and non-enzymatic antioxidants (such as GSH and T-AOC) to remove ROS [40]. It was noted that when the dietary leucine was increased to 0.50%, the GSH-Px, T-AOC, SOD and CAT activities were markedly raised in serum, liver and *longissimus dorsi* muscle tissue. Chen et al. [41]. showed similar results. Thus, the addition of 0.5% dietary leucine is beneficial to the alleviation of oxidative stress caused by heat stress.

Keap1-NRF2 is an important endogenous protective mechanism that regulates the expression of antioxidant enzymes and plays a protective role in the adaptation and survival of cells under heat stress [42]. NRF2 usually binds to Keap1 and is isolated into the cytoplasm as a stable complex. The newly synthesized NRF2 is transferred freely to the nucleus and binds with antioxidant reaction elements to drive the expression of NQO1, SOD and GSH-Px antioxidant genes [42]. In this study, leucine increased the mRNA expression levels of liver NRF2 and its downstream gene NQO1, and decreased the expression of Keap1, suggesting that leucine could alleviate oxidative stress in finishing pigs and the Keap1-NRF2 pathway was involved.

In heat stress environment, the excessive accumulation of ROS is related to the development of muscle atrophy. By increasing the expression of muscle atrophy-related genes Atrogen-1 (MAFbx) and Muscle-specific RING-finger protein 1(MuRF1), the increase of ROS is also related to the decrease of muscle strength and the decrease of mitochondrial function [43,44]. MyoD and MyoG are myogenic markers whose expression is decreased when muscle fibers are damaged [45]. The results of this study showed that 0.50% leucine significantly increased the relative mRNA expressions levels of MyoD and MyoG, and decreased the value of MAFbx and in *longissimus dorsi* muscle tissue of the finishing pigs. Zhang et al. ‘s experimental results on myoblast showed that leucine could be significantly increased the mRNA and protein levels of MyoG and MyoD [46]. In addition, branched amino acids reduce the expression of MAFbx and MuRF1 in soleus muscle by mTOR [47]. The modulation of skeletal muscle mass depends on the balance between protein synthesis and degradation, suggest that leucine may improve muscle mass through mTOR-MAFbx signaling pathway. We also investigated the effects of leucine on the expression levels of skeletal muscle fiber type. Our data showed that leucine up-regulated the relative expression levels of MyHC I and MyHC IIa, and down-regulated the value of MyHC IIx and MyHC IIb. It is speculated that under heat stress environment, adding 0.50% leucine in the diet could alleviate muscle damage caused by heat stress and improve meat quality.

**Figure 5:**
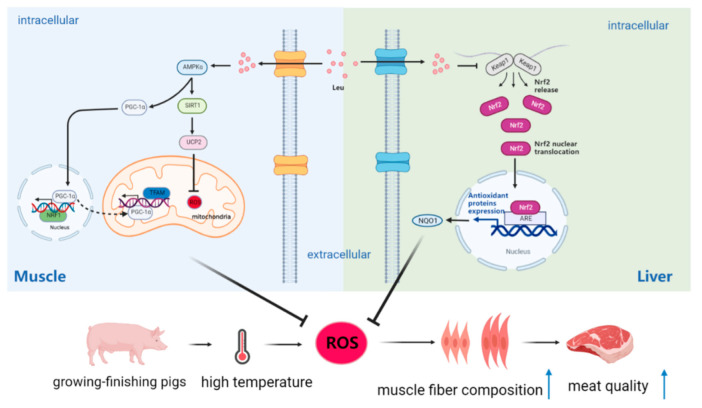
Mechanism of leucine regulating meat quality of finishing pigs under heat stress. Leucine enters muscle cells and activates the gene expression levels of AMPKα, AMPKα activates the expression level of SIRT1, which then activates the expression of UCP2, thereby inhibiting the production of mitochondrial ROS. In addition, AMPK also activates PGC-1α, which entering the nucleus to promote the expression of NRF1 transferred to mitochondria to promote the expression of TFAM and improve the muscle injury caused by heat stress, then improve the meat quality. Leucine enters liver cells and promotes the separation of keap1-NRF2. When NRF2 is released, it was transferred to the nucleus and combined with ARE to activate the expression level of downstream NQO1 gene, inhibiting the production of ROS, and then alleviate the oxidative stress caused by high temperature, ultimately improving the meat quality.

PGC-1α is controlled by regulatory factors related to mitochondrial energy metabolism, such as AMPK and SIRT1. SIRT1 is a deacetylase that acts through activated AMPK to increase phosphorylation of AMPK, resulting in weak oxidative stress response [48]. Chen’s research evidence that leucine induces slow-twitch muscle fibers expression and improves mitochondrial function through AMPK/SIRT1 signaling pathway in porcine skeletal muscle satellite cells. Our results also showed that leucine increased SIRT1 and AMPK expression level under high temperature environment. Uncoupling protein 2 (UCP2) is an intramembrane mitochondrial protein, which is considered as a direct target of PGC-1α transcriptional regulation [49]. UCP2 is involved in the reduction of ROS production and clearance of mitochondrial ROS [50]. Interestingly, leucine significantly enhanced UCP2 mRNA expression levels in the present experiment. In addition, PGC-1α regulates mitochondrial biogenesis by interacting with transcription factors such as NRF1 and mitochondrial transcription factor A (TFAM) [51]. In this study, the mRNA expression level of PGC-1α was upregulated by leucine, and the value of NRF1 was also increased, conjecturing that leucine might ameliorate mitochondrial function damage caused by heat stress through the PGC-1α-NRF1 pathway. Overall, compared to 0.25% group, 0.50% leucine could alleviate muscle injury and keep mitochondrial function through AMPK-TFAM pathways (Figure 5).

## 5. Conclusions

In summary, dietary 0.25% and 0.50% leucine supplementation could improve the growth performance, carcass traits and meat quality of the finishing pigs under heat stress through enhancing the antioxidant capacity, the mRNA expression levels of the genes related to mitochondria function and muscle fiber were increased, for which the effect of 0.50% leucine supplementation is superior, and the Keap1-NRF2 and AMPKα-TFAM pathways might be involved. In pig production, 0.50% leucine could be used to ameliorate heat stress. Further tests are needed to determine whether higher leucine levels are better.

## Figures and Tables

**Table 1:** Ingredients and nutritional composition of basic diets (low protein diet).

Ingredients (%)	Leucine Levels %		
	0	0.25	0.50
Corn	79.42	80.00	80.36
Soybean meal	14.80	13.88	13.20
Wheat bran	3.00	3.00	3.00
Lysine	0.28	0.30	0.32
Methionine	0.00	0.01	0.02
Threonine	0.07	0.08	0.09
Tryptophan	0.01	0.02	0.02
Leucine	0.00	0.25	0.50
Isoleucine	0.00	0.02	0.02
Valine	0.00	0.01	0.03
CaHPO_4_	0.60	0.60	0.60
Limestone	0.52	0.53	0.54
Salt	0.30	0.30	0.30
Premix ^1^	1.00	1.00	1.00
Total	100.00	100.00	100.00
Nutrient content (%)			
Metabolic Energy, ME (MJ/kg) ^2^	12.73	12.70	12.66
Crude Protein	13.58	13.69	13.88
SID Lysine ^2^	0.74	0.73	0.73
SID (Methionine + Cysteine)	0.42	0.42	0.42
SID Threonine	0.47	0.47	0.47
SID Tryptophan	0.13	0.14	0.13
SID Leucine	1.12	1.34	1.57
Total Ca	0.51	0.46	0.48
Total P	0.59	0.50	0.56

^1^ Supplied per kg of diet: vitamin A, 6000 IU; vitamin D3, 4000 IU; vitamin E, 40 IU; vitamin K3, 4 mg; vitamin B1, 6 mg; vitamin B2, 12 mg; vitamin B6, 6 mg; vitamin B12, 0.05 mg; biotin, 0.2 mg; folic acid, 2 mg; niacin, 50 mg; D-calcium pantothenate, 25 mg; Cu (as copper sulfate), 150 mg; Fe (as ferrous sulfate), 100 mg; Mn (as manganese oxide), 40 mg; Zn (as zinc oxide), 100 mg; I (as potassium iodide), 0.5 mg; and Se (as sodium selenite), 0.3 mg. ^2^ Calculated value for ME and SID amino acids. SID: standardized ileal digestible.

**Table 2:** Primers used for quantitative real-time PCR.

Genes ^1^	Primers	Sequences (5′ to 3′)	Product Size, bp
NRF2	Forward	GAAAGCCCAGTCTTCATTGC	121
	Reverse	TTGGAACCGTGCTAGTCTCA	
Keap1	Forward	GCCTCATCGAGTTCGCTTAC	105
	Reverse	CACGGACCACACTGTCAATC	
NQO1	Forward	GTATCCTGCCGAGACTGCTC	134
	Reverse	TAGCAGGGACTCCAAACCAC	
MyHC I	Forward	GGCCCCTTCCAGCTTGA	114
	Reverse	TGGCTGCGCCTTGGTTT	
MyHC IIa	Forward	TTAAAAAGCTCCAAGAACTGTTTCA	136
	Reverse	CCATTTCCTGGTCGGAACTC	
MyHC IIb	Forward	CACTTTAAGTAGTTGTCTGCCTTGAG	80
	Reverse	GGCAGCAGGGCACTAGATGT	
MyHC IIx	Forward	AGCTTCAAGTTCTGCCCCACT	76
	Reverse	GGCTGCGGGTTATTGATGG	
MyoD	Forward	AAGTCAACGAGGCCTTCGAG	123
	Reverse	GGGGGCCGCTATAATCCATC	
MyoG	Forward	AGGCTACGAGCGGACTGA	123
	Reverse	GCAGGGTGCTCCTCTTCA	
MAFbx	Forward	CCCTCTCATTCTGTCACCTTG	104
	Reverse	ATGTGCTCTCCCACCATAGC	
MuRF1	Forward	AGCACGAAGACGAGAAAATC	150
	Reverse	TGCGGTTACTCAGCTCAGTC	
PGC-1α	Forward	CCCGAAACAGTAGCAGAGACAAG	111
	Reverse	CTGGGGTCAGAGGAAGAGATAAAG	
SIRT1	Forward	ACTCTCCCTCTTTTAGACCAAGC	149
	Reverse	AAACCTGGACTCTCCATCGG	
NRF1	Forward	CCTTGTGGTGGGAGGAATGTT	152
	Reverse	AGTATGCTGGCTGACCTTGTG	
TFAM	Forward	GGTCCATCACAGGTAAAGCTGAA	167
	Reverse	ATAAGATCGTTTCGCCCAACTTC	
UCP2	Forward	CTTCTGCGGTTCCTCTGTGT	260
	Reverse	CATAGGTCACCAGCTCAGCA	
AMPKα	Forward	GCATAGTTGGGTGAGCCACA	105
	Reverse	CCTGCTTGATGCACACATGA	
GAPDH	Forward	ACTCACTCTTCTACCTTTGATGCT	123
	Reverse	TGTTGCTGTAGCCAAATTCA	

^1^ NRF2: nuclear factor E2-related factor 2, Keap1: Kelch-like ECH-associated protein-1, NQO1: NAD (P)H: quinone oxidoreductase 1, MyHC I: myosin heavy chain I, MyHC IIa: myosin heavy chain IIa, MyHC IIx: myosin heavy chain IIx, MyHC IIb: myosin heavy chain Iib, MyoD: Myogenic Differentiation Antigen, myoG: myoglobin, MAFbx: muscle atrophy F-box,MuRF1: Muscle RING finger 1, PGC-1α: peroxlsome proliferator—activated receptor-γ coactlvator-1α, SIRT1: Silent information regulator 1, NRF1: nuclear factor E2-related factor 1, TFAM: Recombinant Transcription Factor A, Mitochondrial, UCP2: uncoupling protein 2, AMPKα: adenine monophosphate activated protein kinase α.

**Table 3:** Growth performance of finishing pigs fed the diets with various levels of Leucine.

Item ^1^	Leucine Levels %			SEM	*p*-Value
	0	0.25	0.50		
Initial weight, kg	69.57	67.76	67.57	1.74	0.67
Final weight, kg	88.70	93.23	91.58	1.56	0.07
ADG, Kg·d^−1^	0.42 ^b^	0.61 ^a^	0.59 ^a^	0.04	<0.01
ADFI, Kg·d^−1^	2.26	2.46	2.24	0.16	0.55
F/G	3.95 ^a^	3.20 ^b^	3.17 ^b^	0.16	<0.01

^a,b^ Different superscript letters on the same line are significant differences (*p* < 0.05). ^1^ ADG: average daily weight gain, ADFI: average daily feed intake, F/G: the ratio of feed intake to body weight gain.

**Table 4:** Carcass trait of finishing pigs fed the diets with various levels of leucine.

Item	Leucine Levels %			SEM	*p*-Value
	0	0.25	0.5		
Carcass weight, kg	55.87	58.82	58.92	1.73	0.38
Slaughter rate, %	64.27	65.77	65.62	0.04	0.43
Carcass straight length, cm	87.11	87.86	89.41	1.16	0.39
Carcass slant length, cm	71.76 ^b^	74.90 ^a^	75.03 ^a^	0.10	0.09
Average backfat thickness, mm	20.69 ^a^	16.34 ^b^	16.29 ^b^	1.07	0.01
Loin-eye area, cm^2^	30.06 ^b^	34.30 ^a,b^	36.22 ^a^	1.54	0.03
Lean mass percentage, %	59.78	60.66	61.07	0.52	0.22
Total fat rate percentage, %	15.67	14.00	15.57	0.52	0.21

^a,b^ Different superscript letters on the same line are significant differences (*p* < 0.05).

**Table 5:** Effects of dietary leucine on meat quality of finishing pigs.

Item	Leucine Levels %			SEM	*p*-Value
	0	0.25	0.50		
After slaughter 45 min					
pH	6.51	6.51	6.48	0.04	0.80
L*	47.08	47.54	47.78	0.54	0.65
a*	13.97	13.86	13.77	0.15	0.64
b*	5.66	5.67	5.82	0.15	0.72
After slaughter 24 h					
pH	5.23 ^b^	5.28 ^a,b^	5.34 ^a^	0.03	0.03
L*	55.44	55.92	56.23	0.74	0.75
a*	14.85	14.81	14.75	0.23	0.96
b*	8.10	8.14	8.41	0.22	0.57
Drip loss (%)	28.93 ^a^	23.44 ^b^	23.43 ^b^	0.82	<0.01
Shear force (N)	47.23 ^a^	42.06 ^b^	37.60 ^b^	1.63	<0.01

^a,b^ Different superscript letters on the same line are significant differences (*p* < 0.05).

**Table 6:** Effects of dietary leucine on chemical composition of the muscle tissue.

Item ^1^	Leucine Levels %			SEM	*p*-Value
	0	0.25	0.50		
*Longissimus dorsal* muscle					
CP %	15.81	16.70	16.39	0.67	0.56
IMF %	2.24	2.21	2.15	0.16	0.92
IMP	1.84	2.00	2.04	0.14	0.58
*Biceps femoris* muscle					
CP %	16.07	16.13	16.38	0.82	0.96
IMF %	1.79 ^b^	2.66 ^a^	2.10 ^b^	0.17	<0.01
IMP	2.11 ^b^	2.56 ^a^	2.19 ^b^	0.104	0.01

^a,b^ Different superscript letters on the same line are significant differences (*p* < 0.05). ^1^ IMF, intramuscular fat; IMP, inosine monophosphate, CP: crude protein.

**Table 7:** Effects of different levels of leucine on serum antioxidant enzyme activities of finishing pigs.

Item ^1^	Leucine Levels %			SEM	*p*-Value
	0	0.25	0.50		
GSH-Px, U·mL^−1^	128.10	139.81	141.35	5.16	0.16
T-AOC, U·mL^−1^	0.87 ^b^	0.89 ^ab^	0.90 ^a^	0.012	0.08
SOD, U·mL^−1^	109.90 ^b^	140.51 ^a^	143.40 ^a^	8.13	0.01
CAT, U·mL^−1^	10.28 ^c^	13.83 ^a^	11.91 ^b^	0.39	<0.01
MDA, nmol·mL^−1^	9.60 ^a^	6.19 ^c^	8.18 ^b^	0.38	<0.01

^a–c^ Different superscript letters on the same line are significant differences (*p* < 0.05). ^1^ GSH-Px: Glutathione peroxidase, T-AOC: Total antioxidant capacity, SOD: Superoxide dismutase, CAT: Catalase, MDA: Malondialdehyde.

**Table 8:** Effects of different levels of leucine on liver antioxidant enzyme activities of finishing pigs.

Item ^1^	Leucine Levels %			SEM	*p*-Value
	0	0.25	0.50		
GSH-Px, U·mL^−1^	128.10	139.81	141.35	5.16	0.16
T-AOC, U·mL^−1^	0.87 ^b^	0.89 ^a,b^	0.90 ^a^	0.012	0.08
SOD, U·mL^−1^	109.90 ^b^	140.51 ^a^	143.40 ^a^	8.13	0.01
CAT, U·mL^−1^	10.28 ^c^	13.83 ^a^	11.91 ^b^	0.39	<0.01
MDA, nmol·mL^−1^	9.60 ^a^	6.19 ^c^	8.18 ^b^	0.38	<0.01

^a–c^ Different superscript letters on the same line are significant differences (*p* < 0.05). ^1^ GSH-Px: Glutathione peroxidase, T-AOC: Total antioxidant capacity, SOD: Superoxide dismutase, CAT: Catalase, MDA: Malondialdehyde.

**Table 9:** Effects of different levels of leucine on *Longissimus dorsal* muscles antioxidant enzyme activities of finishing pigs.

Item ^1^	Leucine Levels %			SEM	*p*-Value
	0	0.25	0.50		
GSH-Px, U·mL^−1^	148.01 ^b^	164.22 ^a,b^	177.94 ^a^	7.17	0.04
T-AOC, U·mL^−1^	0.88 ^b^	0.92 ^a^	0.92 ^a^	0.01	<0.01
SOD, U·mL^−1^	136.80 ^b^	172.24 ^a^	156.96 ^a,b^	6.88	<0.01
CAT, U·mL^−1^	16.12 ^b^	16.44 ^a,b^	17.36 ^a^	0.34	0.05
MDA, nmol·mL^−1^	10.79 ^a^	8.06 ^b^	8.83 ^b^	0.48	<0.01

^a,b^ Different superscript letters on the same line are significant differences (*p* < 0.05). ^1^ GSH-Px: Glutathione peroxidase, T-AOC: Total antioxidant capacity, SOD: Superoxide dismutase, CAT: Catalase, MDA: Malondialdehyde.

## Data Availability

The data presented in this study are available in the article.
